# Supplementary material for: Dynein Light Chain Protein Tctex1: A Novel Prognostic Marker and Molecular Mediator in Glioblastoma
Source: Cancers (Basel). 2021 May 27;13(11):2624. doi: 10.3390/cancers13112624 (PMC8199143; doi:10.3390/cancers13112624)
Supplement: Supplementary file 1 [file cancers-13-02624-s001.zip › cancers-1170440-supplementary.pdf]

Figure S1

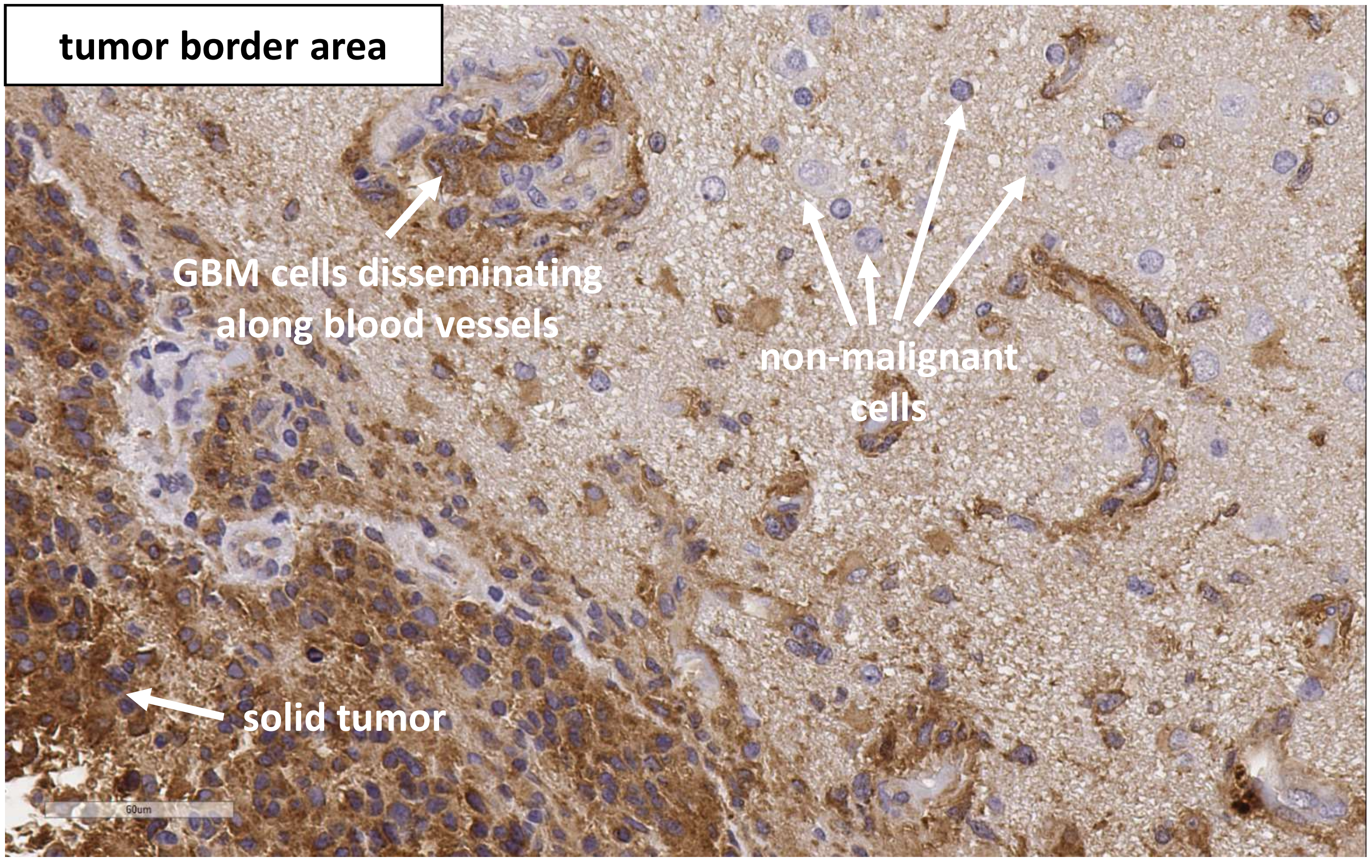
tumor border area
GBM cells disseminating along blood vessels
non-malignant cells
solid tumor
60um

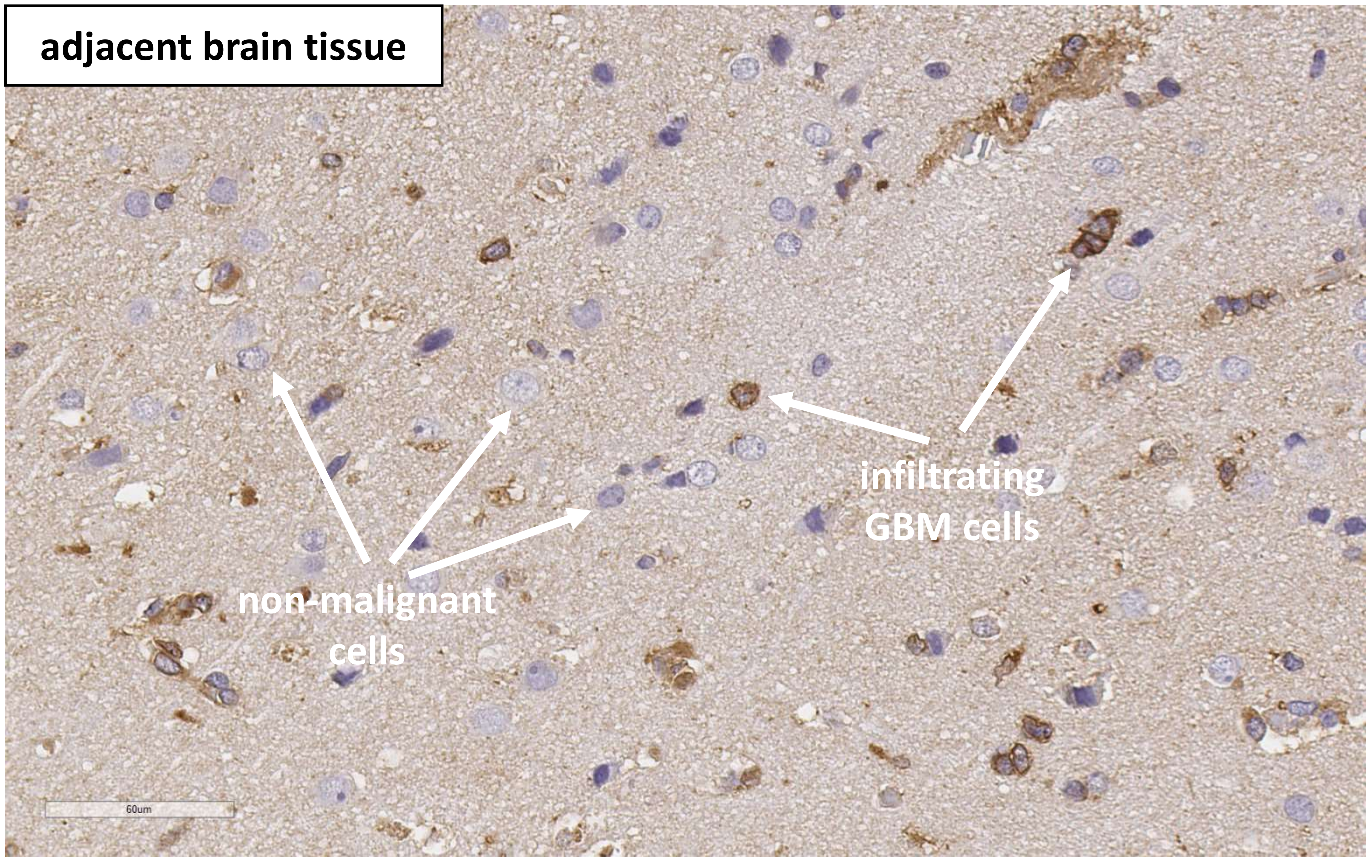
adjacent brain tissue
infiltrating GBM cells
non-malignant cells
60um

Figure S2

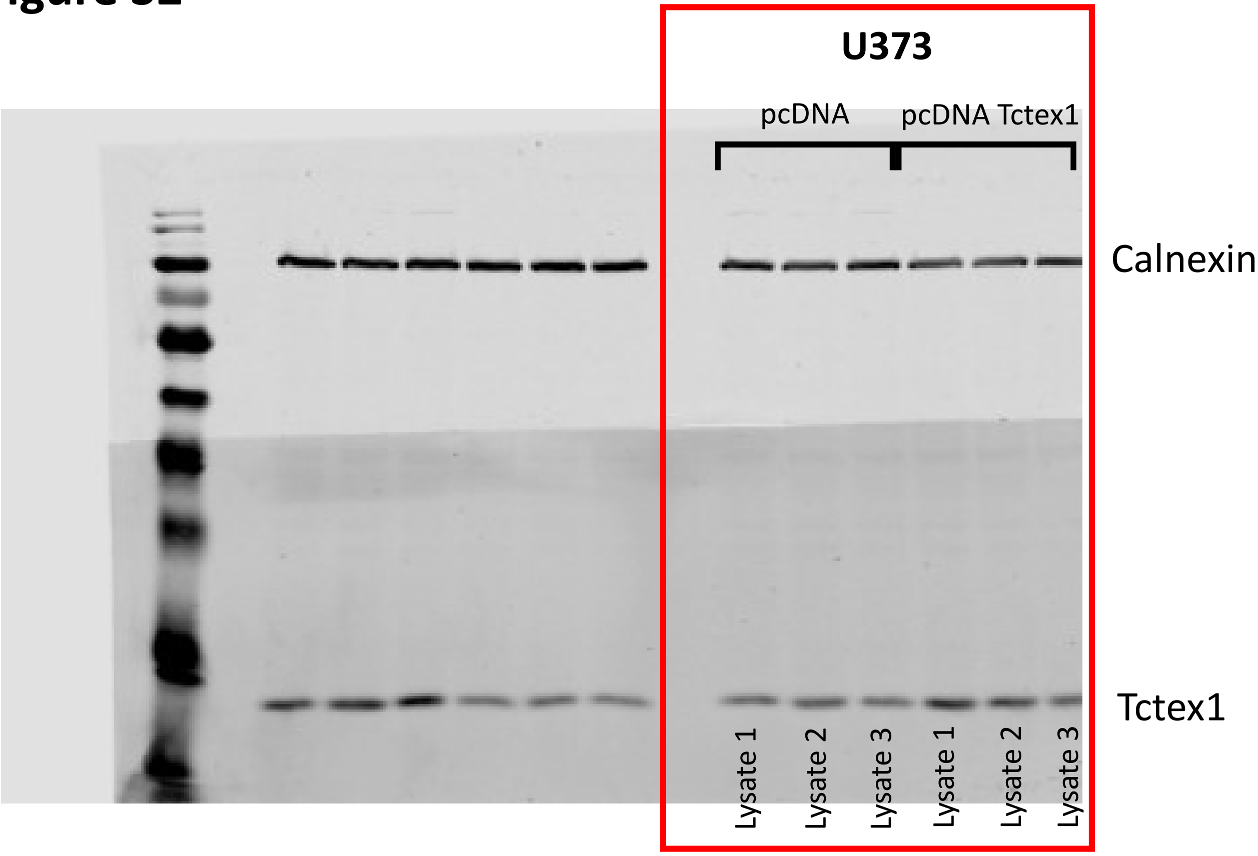

Densitometry intensity ratio

	Tctex1/Calnexin Ratio	Mean
U373 pcDNA – Lysate 1	0,005604	
U373 pcDNA – Lysate 2	0,007853	0,006209
U373 pcDNA – Lysate 3	0,005169	
U373 pcDNA Tctex1 – Lysate 1	0,011084	
U373 pcDNA Tctex1 – Lysate 2	0,009141	0,008329
U373 pcDNA Tctex1 – Lysate 3	0,004763	

Figure S3

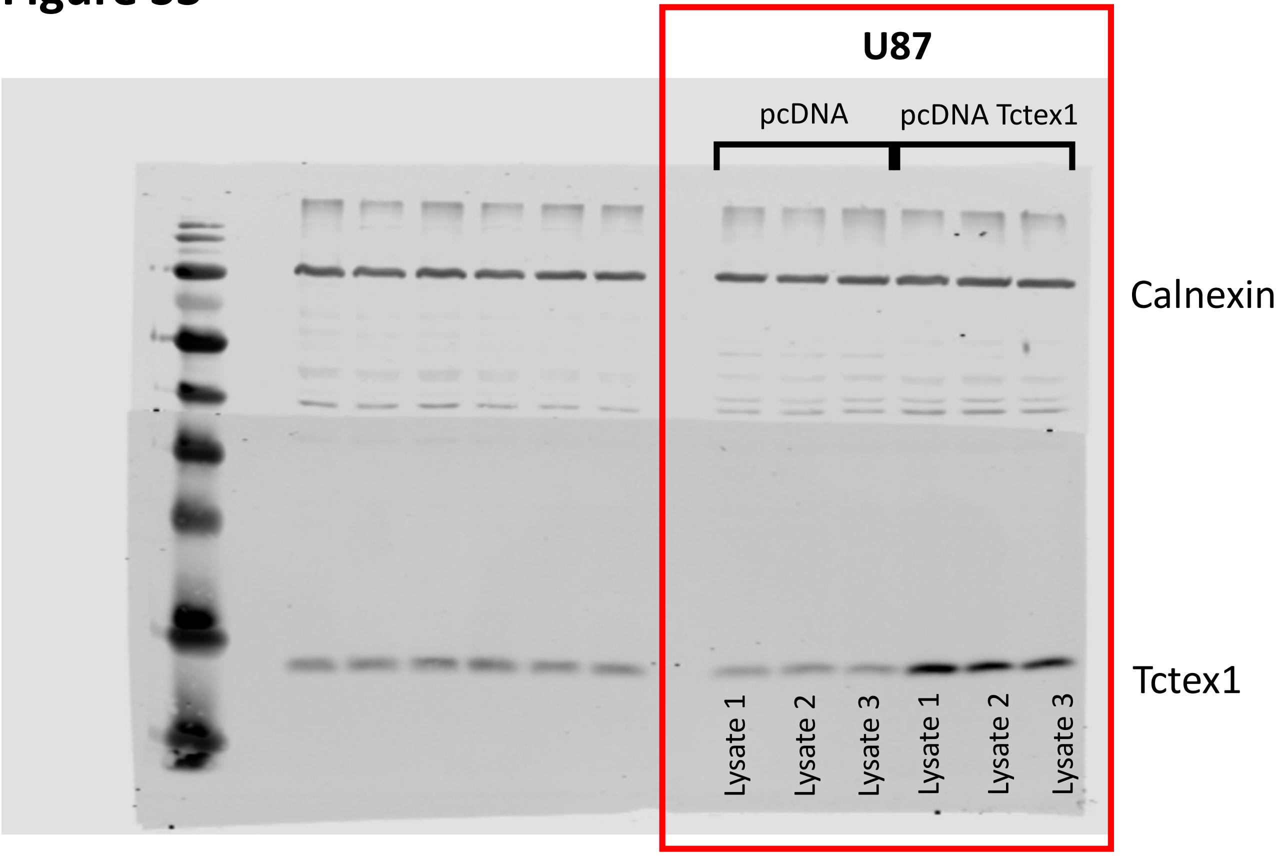

Densitometry intensity ratio

	Tctex1/Calnexin Ratio	Mean
U87 pcDNA – Lysate 1	0,021752	
U87 pcDNA – Lysate 2	0,031955	0,024482
U87 pcDNA – Lysate 3	0,019739	
U87 pcDNA Tctex1 – Lysate 1	0,085385	
U87 pcDNA Tctex1 – Lysate 2	0,053856	0,06306
U87 pcDNA Tctex1 – Lysate 3	0,049938	
